# Quality of Internet Videos Related to Pediatric Urology in Mainland China: A Cross-Sectional Study

**DOI:** 10.3389/fpubh.2022.924748

**Published:** 2022-06-15

**Authors:** Gaochen Bai, Kai Fu, Wen Fu, Guochang Liu

**Affiliations:** ^1^Guangzhou Institute of Pediatrics, Guangzhou Women and Children's Medical Center, Guangzhou Medical University, Guangzhou, China; ^2^Department of Pediatric Urology, Guangzhou Women and Children's Medical Center, Guangzhou Medical University, Guangzhou, China

**Keywords:** quality, internet, pediatric urology, Chinese video, COVID-19

## Abstract

**Background:**

Pediatric urological diseases pose serious threats to children's physical and mental health. The COVID-19 pandemic has resulted in poor pediatric outcomes for cryptorchidism, hypospadias, and testicular torsion. Presently, many people tend to seek health information via social media platforms. This study aims to quantitatively assess the quality of videos as an information source for pediatric urology in mainland China.

**Methods:**

In this cross-sectional study, a search was performed on social media platforms (Tiktok, Bilibili, and Weibo) with the search terms “cryptorchidism”, “hypospadias”, and “testicular torsion”. The first 30 results with any search term listed by relevance were selected in each platform. Video features (duration, number of likes, comments, and shares) and video sources were collected. Each video included in the study was assessed using DISCERN, Journal of the American Medical Association (JAMA) Benchmark Criteria, and Hexagonal Radar Schema. A correlation analysis was performed considering video features, video source, DISCERN scores and JAMA scores.

**Results:**

A total of 152 videos were included and analyzed. The majority of videos were from physicians (65.8%). According to the DISCERN classification, most videos were rated as “very poor” (48.0%) and “poor” (36.8%). The mean DISCERN and JAMA scores were 36.56 and 2.68, respectively. The duration of videos uploaded by physicians was the shortest (*P* < 0.001). The video source had no relevance to numbers of “likes”, “comments”, “shares”, DISCERN scores, and JAMA scores (all *P*-values > 0.05). Other than video duration (*P* < 0.001), there was no statistically significant difference between groups for any of the recorded or calculated video data (all *P* values > 0.05). Hexagonal Radar Charts showed the severe imbalance and deficiency of video information. In general, Tiktok videos with the shortest duration received the most numbers of “likes”, “comments”, and “shares”, whereas the overall quality of videos on Weibo was relatively high.

**Conclusions:**

Despite most of the videos on social media platforms being uploaded by medical authors, the overall quality was poor. The misleading, inaccurate and incomplete information may pose a health risk to the viewers, especially during the COVID-19 pandemic. Much effort needs to be undertaken to improve the quality of health-related videos regarding pediatric urology.

## Introduction

Pediatric urological diseases pose serious threats to the physical and mental health of children. Cryptorchidism, hypospadias, and testicular torsion are three common urological diseases for newborns and children, with incidence rates of 90–100/10,000, 20.9/10,000, and 2.5/10,000, respectively (1–3). It is worth noting that public awareness of these diseases is poor, which leads to recurring inaccuracy in identification and delays in surgery. Previous studies reported that only 16.9% of children with cryptorchidism and 14.5% of children with hypospadias received timely surgery based on the European Association of Urology (EAU) Guidelines. The orchiectomy rate was 42% in boys undergoing surgery for testicular torsion due to delayed management (4–7).

Since the COVID-19 infection and global spread, social distancing and reduced travel have been strictly advised to control the disease spreading (8). Usage of medical resources has also been affected, primarily by increased critically ill patients and overcrowding of medical facilities (9). Reduced hospital visits and delays in hospitalization and operations for pediatric patients have been reported due to the parents' hesitancy for symptoms not related to COVID-19, which may lead to more complications and poor outcomes (10–12). Such a fearful environment prompts people to access health information and try identifying early symptoms of the disease online.

The internet has developed rapidly and become an essential approach for obtaining and disseminating health information (13, 14). In Hong Kong, China, 87.44% of primary care patients had used the internet to find health information, and 65.97% searched once monthly or more (15). The internet may facilitate and develop public knowledge of health. Furthermore, most social media users for health communications were 11–34 years old, with more female than male users of social network sites (16). These frequent users are just the population at risk of pediatric urological diseases or their parents. Today, social media platforms enable sharing attractive videos regarding health information with large personal networks and the public. The illustrated and documented information can be easier to process and remember than plain text, eliciting affective reactions and motivating consumers' health responses (17, 18).

However, social media platforms may contain misleading or poor-quality information due to the absence of any regulations or restrictions on video content from any source (19, 20). Moreover, health information is constantly updated, so the information provided by videos on social media platforms may be out of date (21). Such inaccurate and unreliable health information can affect health popularization and public disease management.

In mainland China, there are many kinds of social media platforms. Tiktok, Bilibili, and Weibo have become increasingly popular and essential channels for health information dissemination for Chinese people, with user-friendly experiences on computers, tablets, and smart mobile phones (22–24). There are many health-related videos on these platforms, but the literature lacks a quantitative quality assessment of video content related to health information, including pediatric urological diseases. This study aims to assess the quality of internet videos as an information source for cryptorchidism, hypospadias, and testicular torsion.

## Materials and Methods

### Recruitment

In this cross-sectional study, a search was performed on social media platforms (i.e., Tiktok, Bilibili, and Weibo) from March 29th to March 31th, 2022. The search terms were “隐睾” (cryptorchidism), “尿道下裂” (hypospadias), and “睾丸扭转” (testicular torsion). The search history was deleted before searching to reduce any impact on the search results and outcomes. The first 30 results with any search term listed by relevance were selected in each platform (default search setting). Commercial videos, non-Mandarin videos, videos without audio, irrelevant videos, and duplicate videos were excluded.

### Collection of Video Features and Source

Video features assessed include total video duration, sources, number of days online, number of “likes”, number of comments, and number of shares. The authors defined video sources as physicians, patients or guardians, medical institutes, health educators, news media, and independent users.

### Assessment of Quality

DISCERN, JAMA Benchmark Criteria, and Hexagonal Radar Schema were used for quality analyses of the videos (22, 25, 26). The estimate of specialized medical issues related to pediatric urology was based on the EAU guidelines (6).

DISCERN consisted of 16 questions in total, with each question scored from 1 to 5 points. Questions were divided into three sections: reliability (questions 1–8), quality information about treatment options (questions 9–15), and overall score (question 16). The total DISCERN score was calculated by summing up scores over questions 1–15. All videos were divided into five categories based on their total DISCERN score: very poor (<27), poor (27–36), fair (39–50), good (51–62), and excellent (63–75).

JAMA benchmark criteria were used to evaluate online health information reliability, including four criteria (authorship, attribution, disclosure, and currency). Each satisfied criterion counted 1 point, with a maximal score of 4 points.

Hexagonal Radar Schema is a coded scale that reflects videos' six dimensions, including the definition, signs, risk factors, examinations, management, and disease outcomes. According to the specific criteria and examples, points (from 1 to 4) would be assigned at each dimension based on the evaluation of videos. The spotlight and weight of each video could be visually presented by the shape and size of the radar chart. Any dimension scoring more than one point in this chart would be acceptably clear.

The detailed information for DISCERN, JAMA Benchmark Criteria, and Hexagonal Radar Schema are available online as Supplementary Material.

Two independent urologists (GB and KF) evaluated all videos. Any discrepancies between evaluators were resolved by discussion with a third author (GL) for consensus.

### Ethics Statement

This study focused on the quality assessment of videos contributed and viewed by the public on social media platforms, so ethics committee approval was not required.

### Statistical Analysis

Statistical analyses were conducted using GraphPad Prism Software 8.3 (La Jolla, California, USA). Categorical variables are presented as frequency and ratios (%), and continuous variables are presented as mean ± standard deviation (SD) and median (interquartile range, IQR). The Kruskal-Wallis test and Bonferroni adjustment determined statistically significant differences between more than two groups of any independent variable. A *P*-value < 0.05 was considered statistically significant.

## Results

### Video Features and Quality Assessment

**Figure 1:**
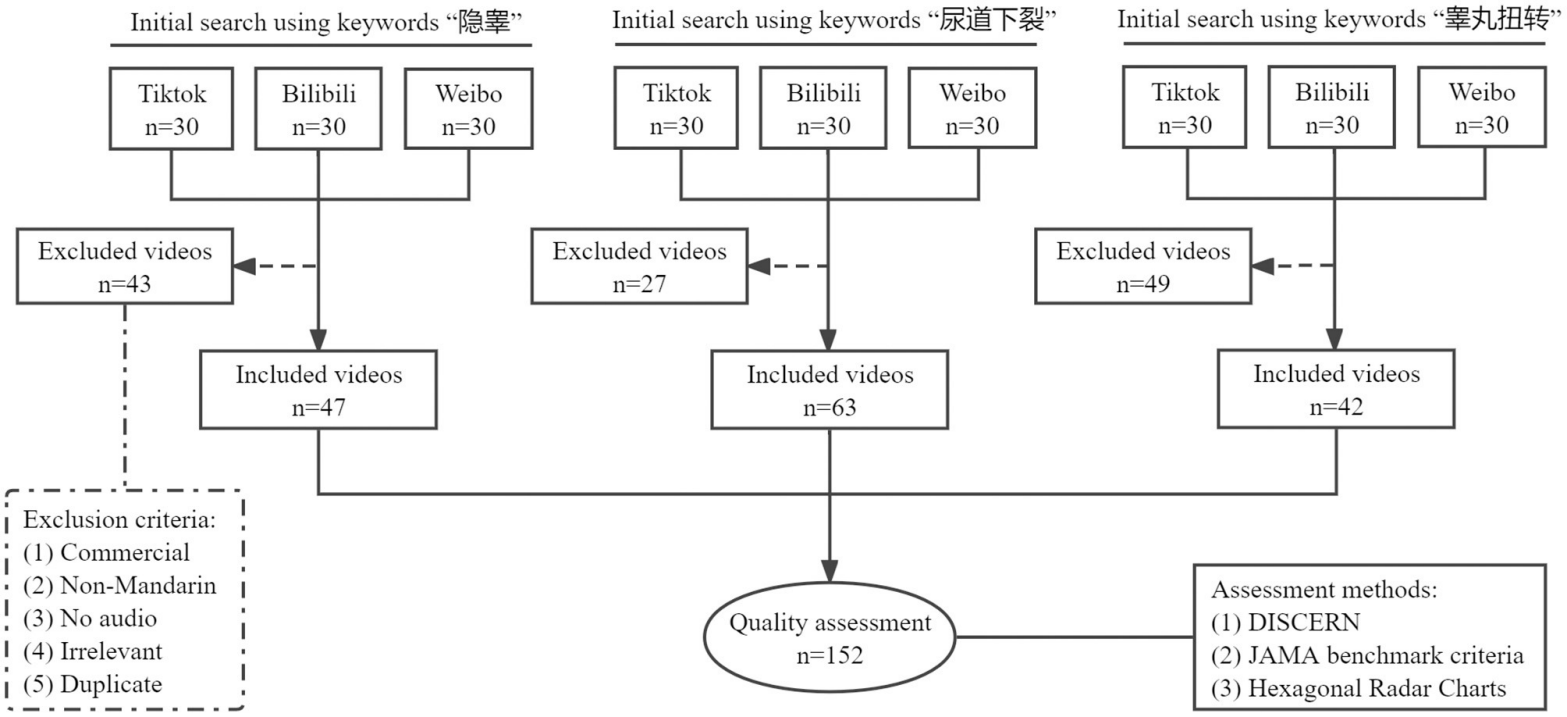
Flowchart of the selection of internet videos for analysis.

**Figure 2:**
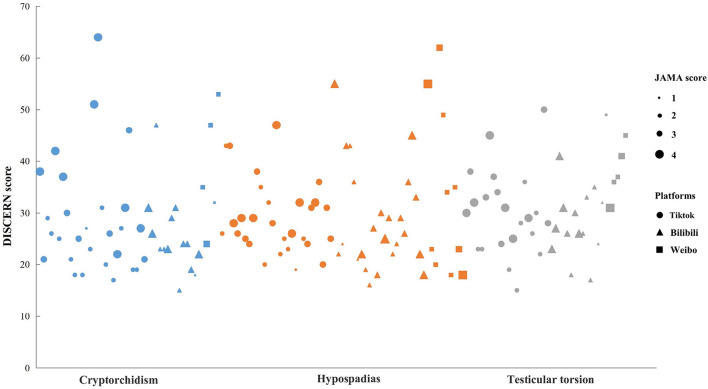
Detailed results of DISCERN and JAMA scores of each included video.

In the keyword search on social media platforms (i.e., Tiktok, Bilibili, and Weibo), ninety videos for each disease entered the initial review. After screening according to the exclusion criteria, a total of 152 videos were included and analyzed for this study (Figure 1). The general features of the 152 videos are presented in Table 1. The mean DISCERN total score was 29.60 ± 9.77 [median (IQR): 27 (23, 34.75)], and the mean JAMA score was 2.74 ± 0.90 [median (IQR): 3 (2, 3.75)]. Figure 2 shows the detailed results of DISCERN and JAMA scores of each included video.

**Table 1:** General features of included videos.

**Video features**	**Mean ±SD**	**Median (IQR)**
Duration (s)	97.30 ± 125.38	50.5 (34.25, 99.5)
Number of days online	327.09 ± 312.15	265 (106, 472.5)
Number of likes	2,106.13 ± 11,027.93	72.5 (13, 387.25)
Number of comments	214.51 ± 925.52	8.5 (1, 51.75)
Number of shares	798.09 ± 4,503.51	10.5 (1, 90)
DISCERN reliability	17.57 ± 5.35	17 (14, 20)
DISCERN treatment	12.03 ± 5.25	11 (7, 14.75)
DISCERN quality	2.24 ± 0.91	2 (2, 3)
DISCERN total	29.60 ± 9.77	27 (23, 34.75)
JAMA score	2.74 ± 0.90	3 (2, 3.75)

The data in Table 2 shows that the majority of videos were uploaded by physicians (65.8%), followed by news media (11.2%), independent users (8.6%), medical institutes (7.2%), health educators (6.0%), and patients or guardians (1.3%). Although physicians contributed the most videos, the duration of videos uploaded by them [median (IQR): 43.50 (32, 63)] was the shortest (*P* < 0.001). Although videos uploaded by news media or physicians seemed to receive more “likes”, “comments”, and “shares”, there was no statistical significance. Moreover, videos related to hypospadias were the most numerous (*n* = 63, 41.1%), but testicular torsion seemed to have received more attention from the public.

**Table 2:** General features sorted by video source and diseases.

	**Number of videos, *n* (%)**	**Duration (s), median (IQR)**	**Number of likes, median (IQR)**	**Number of comments, median (IQR)**	**Number of shares, median (IQR)**
**Videos source**					
Physicians	100 (65.8)	43.50 (32, 63)^b^	84.5 (18, 311.75)	10.5 (1, 111)	8.5 (1, 44.5)
Patients or guardians	2 (1.3)	518.5	61.5	17	17.5
Medical institutes	11 (7.2)	79 (35, 131)	16 (3, 472)	6 (0, 104)	3 (1, 49)
Health educators	9 (6.0)	88 (36.5, 118.5)	84 (15, 316)	4 (0, 94)	20 (2.5, 320)
News media	17 (11.2)	90 (60.5, 223.5)	395 (9, 2,296.5)	34 (3, 687)	101 (1.5, 407)
Independent users	13 (8.6)	110 (46, 303)	8 (4, 297)	4 (0, 32)	2 (0, 40.5)
*P-*value^a^	–	<0.001	0.332	0.117	0.270
**Diseases**					
Cryptorchidism	47 (30.9)	33 (53, 79)	95 (16, 318)	10 (3, 67)	17 (1, 118)
Hypospadias	63 (41.4)	51 (37, 95)	19 (3, 84)	1 (0, 16)	2 (0, 11)
Testicular torsion	42 (27.6)	49.5 (31.75, 131.5)	350.5 (96.75, 936)	37.5 (9.5, 106.75)	57.5 (24.25, 290.5)

a *Kruskal-Wallis test*.

b *Compared with news media and independent users, P = 0.007 and =0.049, respectively*.

### Association of the Video Source, Related Diseases, Discern and JAMA Score

The detailed results of questionnaires in DISCERN and JAMA benchmark criteria are highlighted using different color gradations in Supplementary Material, classified by the video source. The Kruskal-Wallis test showed that DISCERN reliability scores, treatment scores, quality scores, total scores, and JAMA scores had no significant correlation with the video source. In other words, videos uploaded by medical personnel or groups were not superior in quality. In addition, there were no significant correlations between the type of diseases and DISCERN/JAMA score (see Table 3).

**Table 3:** Quality assessments according to video source and diseases.

	**DISCERN reliability, median (IQR)**	**DISCERN treatment, median (IQR)**	**DISCERN quality, median (IQR)**	**DISCERN total, median (IQR)**	**JAMA score, median (IQR)**
**Video source**					
Physicians	16 (13, 19)	11 (7.25, 14)	2 (2, 2.75)	26.5 (23, 32)	3 (2, 3)
Patients or guardians	18.5	16.5	3	35	3
Medical institutes	16 (11, 24)	10 (7, 22)	2 (1, 3)	29 (19, 46)	3 (2, 3)
Health educators	19 (15.5, 24)	11 (7, 19)	2 (2, 3.5)	28 (22.5, 43)	4 (2.5, 4)
News media	19 (15.5, 22.5)	12 (10, 15.5)	3 (2, 3)	32 (25.5, 39)	3 (2, 4)
Independent users	17 (11, 19.5)	9 (7, 14.5)	2 (1, 3)	24 (19.5, 34.5)	2 (1.5, 2.5)
*P*-value^a^	0.137	0.356	0.104	0.213	0.087
**Diseases**					
Cryptorchidism	16 (13, 18)	10 (8, 13)	2 (2, 3)	26 (21, 31)	3 (2, 4)
Hypospadias	17 (13, 20)	10 (7, 15)	2 (2, 3)	26 (22, 35)	3 (2, 3)
Testicular torsion	19 (14, 22)	12.5 (9, 14.25)	2 (2, 3)	30 (24.75, 36)	3 (2, 4)
*P*-value^a^	0.203	0.206	0.078	0.177	0.923

a *Kruskal-Wallis test*.

### Evaluation Outcomes of Discern Classification

The DISCERN classification data showed that 48.0% were “very poor”, 36.8% were “poor”, 11.2% were “fair”, 3.2% were “good”, and 0.7% were “excellent”. Other than video duration (*P* < 0.001), there was no statistically significant difference between groups for any of the recorded or calculated video data (see Table 4).

**Table 4:** Distribution of DISCERN classification according to video features, source, and diseases.

**Variable**	**Very poor**	**Poor**	**Fair**	**Good**	**Excellent**	***P*-value^**a**^**
**Video features, median (IQR)**						
Duration (s)	40 (30, 61.5)	51.5 (35.5, 86)	124 (90, 241)	233 (190.5, 349.5)	201	<0.001
Number of likes	47 (5, 207)	140 (14.25, 470.5)	84 (20, 491)	59 (10.5, 2,883.5)	767	0.227
Number of comments	5 (0.5, 28)	12.5 (1, 95)	20 (2, 178)	23 (3, 253.5)	218	0.120
Number of shares	6 (0, 29.5)	33 (1.25, 110)	22 (4, 130.5)	30 (4, 168.5)	481	0.062
**Video source**, ***n***						
Physicians	50	41	6	3	0	
Patients or guardians	1	0	1	0	0	
Medical institutes	5	3	1	1	1	
Health educators	3	2	4	0	0	
News media	5	8	3	1	0	
Independent users	9	2	2	0	0	
**Diseases**, ***n***						
Cryptorchidism	26	14	4	2	1	
Hypospadias	32	21	7	3	0	
Testicular torsion	15	21	6	0	0	
Number of videos, *n* (%)	73 (48.0)	56 (36.8)	17 (11.2)	5 (3.2)	1 (0.7)	

a *Kruskal-Wallis test*.

### Hexagonal Radar Schema of Different Diseases

**Figure 3:**
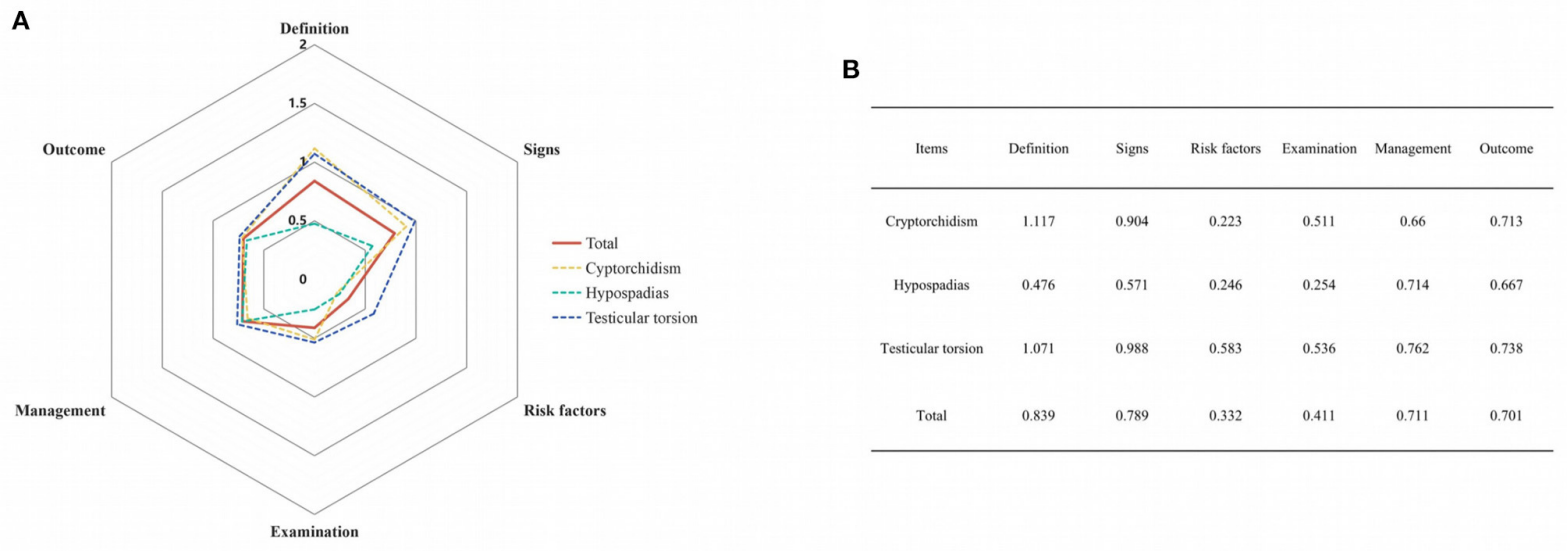
Hexagonal radar charts of included videos. **(A)** Hexagonal Radar Charts of videos on cryptorchidism, hypospadias, and testicular torsion. **(B)** Specific results presented as scores.

The Hexagonal Radar Charts in Figure 3 showed the severe imbalance and deficiency of video information on social media platforms. More than 30% of the videos delivered moderate to high quality content (>1 point) on the definition (*n* = 54, 35.5%), and signs (*n* = 46, 30.3%), but the percentage on risk factors (*n* = 17, 11.2%), examination (*n* = 15, 9.9%) and, outcome (*n* = 22, 14.5%) were relatively low. Other than the definition of cryptorchidism and testicular torsion, the mean points of subjects were all less than one.

### Features and Quality Comparison of Videos on Different Social Media Platforms

**Figure 4:**
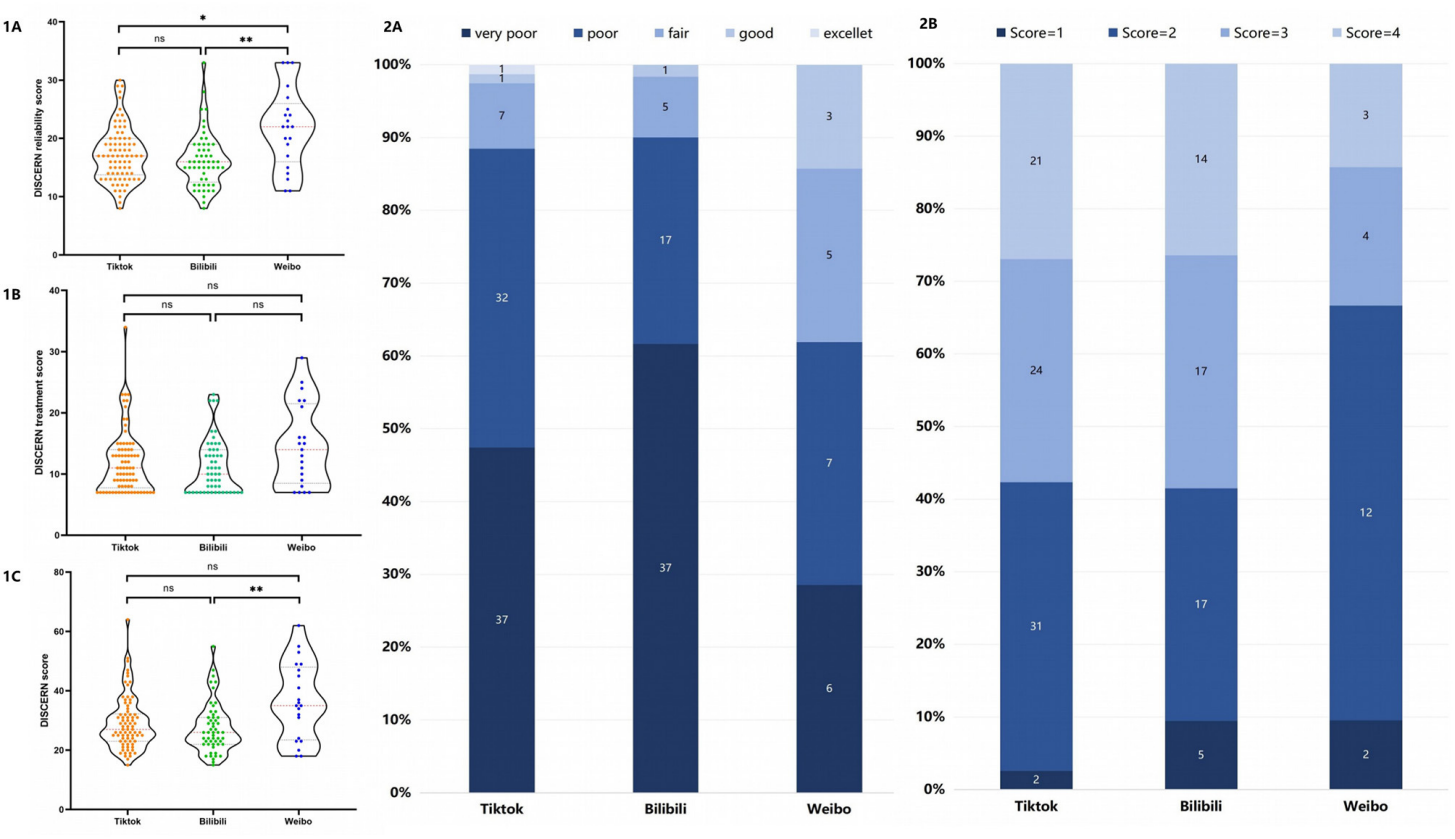
Quality comparison of videos on different social media platforms. **(1A–C)** DISCERN score comparison of videos on different social media platforms. **(2A)** Distributions of DISCERN classification. **(2B)** Distributions of JAMA scores.

The number of videos included and analyzed on Tiktok, Bilibili, and Weibo was 78, 53, and 21. In general, Tiktok's videos with the shortest duration received the most numbers of “likes”, “comments”, and “shares” (see Table 5). The median DISCERN reliability score of videos on Weibo was higher than Tiktok and Bilibili (see Figure 4-1A). The median DISCERN treatment score in these three platforms had no statistical differences (see Figure 4–1B). The median DISCERN total score of videos on Weibo was higher than that on Bilibili (see Figure 4–1C). The distributions of DISCERN classification and JAMA score are shown in Figures 4–2A,B.

**Table 5:** General features of included videos in different social media platforms.

**Platforms**	**Number of videos, *n* (%)**	**Duration (s), median (IQR)**	**Number of likes, median (IQR)**	**Number of comments, median (IQR)**	**Number of shares, median (IQR)**
Tiktok	78 (51.3)	39.5 (31, 62)^b^	177.5 (47, 473.25)^c^	16 (4, 69.25)^d^	31 (6, 156.25)^e^
Bilibili	53 (34.9)	64 (43, 144)	15 (3, 62.5)	1 (0, 17)	1 (0, 13.5)
Weibo	21 (13.9)	95 (67, 195.5)	16 (1, 695)	21 (0.5, 98.5)	26 (0, 77)
*P*-value^a^	–	<0.001	<0.001	<0.001	<0.001

a *Kruskal-Wallis test*.

b *Compared with Bilibili and Weibo, P = 0.001 and < 0.001, respectively*.

c *Compared with Bilibili and Weibo, P < 0.001 and =0.04, respectively*.

d *Compared with Bilibili, P < 0.001*.

e *Compared with Bilibili, P < 0.001*.

## Discussion

With the increasing popularity of the internet, people are accustomed to searching for health information on social media platforms (27). The videos on Tiktok, Bilibili, and Weibo have provided a more intuitive and friendly manifestation for the public in mainland China. However, previous data for pediatric urology has shown that only 22.2% of videos contained information that can also be found in the EAU guidelines, and none of the videos contained any cited evidence to reinforce statements that were being made (28). Moreover, many studies indicated that most videos on social media platforms were of poor quality with misleading and incorrect information, which could have catastrophic implications for healthcare consumers (29). This worrying state of video platforms may easily lead to patient misunderstandings and prevent them from making correct choices and seeking timely treatments. Therefore, it is vital to quantitatively assess the quality of internet videos in mainland China from a pediatric urological perspective, which has not previously been investigated.

When assessing the quality of videos on social media platforms, the selection of assessment instruments directly affects the evaluation results. We chose the DISCERN instrument to focus more on integrity and reliability, both intrinsically important for health information. Authors and viewers often overlook references and content sources, copyright information, and conflicts of interest in videos, so the JAMA benchmark criteria were used to evaluate these critical subjects. In addition, the Hexagonal Radar Schema reflects multiple dimensions of the videos and whether balanced and comprehensive information was provided to the public. At the same time, some data about these videos were collected to illustrate their fundamental characteristics and correlation with the outcomes. The research contents were discussed, and multiple physicians and researchers formulated strategies.

### Major Findings

The mean DISCERN and JAMA scores were 29.60/75 and 2.74/4. According to the DISCERN classification, 60.6% of the videos were of very poor or poor quality, and only 3.9% were assessed as good or excellent quality. The low score rates reflected the videos' poor integrity and reliability regarding pediatric urology on social media platforms. The Hexagonal Radar Charts indicated that, although some aspects (for example, definition and signs) of the disease were discussed relatively more, the mean points of all subjects were lower than one, inferring that videos could not comprehensively explain the definition, signs, risk factors, examinations, management, and outcomes of pediatric urological diseases with acceptably clear quality. Keelan et al. (30) first reported that 38% of analyzed Youtube videos objected to immunization but received a higher mean star rating and more views than those supporting immunization. It was worth noting that 45% of these negative videos conveyed messages that contradicted the reference standards. After that, increasing numbers of studies aroused people's concern for the frequently misleading and poor quality videos on social media platforms. Despite these downsides, the increasing popularity of this video-sharing platform prompts more people to use it to disseminate and acquire health information (29).

Physicians and medical institutes uploaded more than two-thirds of included videos, but the overall quality was poor. Surprisingly, videos from medical specialists and groups did not receive more public attention and higher quality evaluation rates than those from other sources, which was inconsistent with the findings of previous similar studies (22, 28, 31). Such a poor outcome warrants concern: health information provided by specialists and academic institutions does not achieve the expected quality. Moreover, the video duration from physicians is generally shorter than others. From these observations, most physicians were perfectly willing to capture and upload a series of videos by breaking a topic into multiple sections, which may increase the total clicks, likes, and shares much concerned by authors. However, because social media platforms recommend videos to viewers based on algorithms or randomness when searching (32), people may not acquire the complete picture of health information timely and efficiently.

Many viewers are used to clicking on the videos with higher popularity first, hoping for more reliable and comprehensive information from specialized individuals or groups. However, Loeb et al. (33) analyzed 150 internet videos related to bladder cancer and found that the misinformation was present in 29.3% of videos and reached 1,483,859 viewers. Worse, there was a significant positive correlation between the presence of misinformation and views per month. The current study found that the most popular videos did not have the highest quality, the highest valued videos were not the most popular videos, and the number of “likes”, “comments”, and “shares” had no relevance to the video quality score and source. As expected, longer videos tend to get higher DISCERN classifications due to the more comprehensive and detailed content. However, the number of “likes”, “comments”, and “shares” were not correlated with the DISCERN classification, revealing that users may not always view and trust high-quality health information with little discernment. This finding highlights the importance of self-education to identify misleading information for the public accurately. Meanwhile, much effort needs to be undertaken for active recommendations of evidence-based health education materials provided by relatively professional individuals and institutions.

A recent study compared videos regarding erectile dysfunction treatment on YouTube and Tiktok. Despite considerably unreliable information, YouTube videos were of higher quality than Tiktok videos (34). Due to the lack of comparative studies among different social media platforms in mainland China, the current study first analyzed videos from different platforms, indicating that videos on Tiktok were more popular. In contrast, the overall quality of videos on Weibo was relatively high. This is probably because TikTok is a short-video platform and the videos' short duration may lead to incompletion of related health information. Tiktok has accumulated more than 1.1 billion active users and includes almost half of adults younger than 30 years worldwide (35), which necessitates it to provide more high-quality health information to accelerate the progress of public health education.

### Challenges and Expectations

In the era of the COVID-19 pandemic, the role of social media platforms in engaging public health has dramatically grown due to the non-contact and convenience advantages (36). Based on the results of this study, social media platforms have enormous potential to improve the quality of videos related to health information and should be responsible to the public from ethical and legal perspectives.

While it is impractical to establish laws to regulate health information on social media platforms, we should put forward proposals to utilize social media appropriately and positively. Currently, video content from social media platforms is unreliable due to the frequently misleading and incomplete information. One feasible suggestion for eradicating the inaccurate information would be to ask authors to add sources and references in the introduction section of the health-related video, labeling video segments according to the specified standards. Simultaneously, video platforms can provide specific questionnaires for the viewers to assess the video quality and improve the filtering algorithms to prioritize high-quality videos when searching, based on the continuously updated evaluation results. Overwhelmingly, specialists and academic institutions should provide more high-quality, reliable videos that follow clinical practice guidelines to social media platforms. Viewers should be cautious and try to accurately identify the health information related to pediatric urology.

### Limitations

This study has several limitations. Firstly, the search results on social media platforms are dynamic over time, and the data collected and analyzed only represents one point in time. Secondly, despite deleting the search history before searching, the research results may differ according to different geographic locations, user habits, or other unknown algorithm restrictions. Thirdly, the analysis of data and comparison among different platforms were limited to the first 30 videos for each keyword, which may lead to biased conclusions. Fourthly, because the function to check the number of views was unavailable on Tiktok and Weibo, there were minimal flaws in data collection and analysis.

## Conclusions

Social media platforms provide a popular and indispensable mechanism for the public to access health information. This study is the first report to assess the quality of videos related to pediatric urology on social media platforms in mainland China. The data revealed that despite most of the videos being uploaded by medical authors, the overall quality was poor. The risks of misleading, inaccurate, and incomplete information cannot be ignored, especially in the era of the COVID-19 pandemic. It is vital to improve the quality of videos regarding pediatric urology and promote self-education amongst the public.

## Data Availability Statement

The raw data supporting the conclusions of this article will be made available by the authors, without undue reservation.

## Author Contributions

GB designed the study, carried out data analysis, and drafted the manuscript. GB and KF participated in data analysis, collected all relevant data, and assisted in study conception and design. WF and GL conceived the study, participated in its design and coordination, and helped draft the manuscript. All authors read and approved the final manuscript.

## Funding

This research was supported by the Research Foundation of Guangzhou Women and Children's Medical Center for Clinical Doctors.

## Conflict of Interest

The authors declare that the research was conducted in the absence of any commercial or financial relationships that could be construed as a potential conflict of interest.

## Publisher's Note

All claims expressed in this article are solely those of the authors and do not necessarily represent those of their affiliated organizations, or those of the publisher, the editors and the reviewers. Any product that may be evaluated in this article, or claim that may be made by its manufacturer, is not guaranteed or endorsed by the publisher.

## Supplementary Material

Supplementary Figure 1 Detailed results of questionnaires for included videos in DISCERN and JAMA benchmark criteria. Each rectangular unit corresponds to an included video with the source and quality assessment based on the titles in the image. Red or blue represent assessment by DISCERN or JAMA benchmark criteria. Different levels of color represent different single question scores or total scores.

Supplementary Table 1 DISCERN scoring system.

Supplementary Table 2 JAMA benchmark criteria.

Supplementary Table 3 Hexagonal charting system.

## Abbreviations

JAMA: Journal of the American Medical Association
IQR: Interquartile range.
