# Supplementary material for: Quality of Internet Videos Related to Pediatric Urology in Mainland China: A Cross-Sectional Study
Source: Front Public Health. 2022 Jun 15;10:924748. doi: 10.3389/fpubh.2022.924748 (PMC9240759; doi:10.3389/fpubh.2022.924748)
Supplement: Supplementary Table 1 — DISCERN scoring system. [file Table_1.DOCX]

| **Question** | | | **Score** |
| --- | --- | --- | --- |
| Section 1 | 1 | Are the aims clear? | 1-5 |
|  | 2 | Does it achieve its aims? | 1-5 |
|  | 3 | Is it relevant? | 1-5 |
|  | 4 | Is it clear what sources of information were used to compile the publication (other than the author or producer)? | 1-5 |
|  | 5 | Is it clear when the information used or reported in the publication was produced? | 1-5 |
|  | 6 | Is it balanced and unbiased? | 1-5 |
|  | 7 | Does it provide details of additional sources of support and information? | 1-5 |
|  | 8 | Does it refer to areas of uncertainty? | 1-5 |
| Section 2 | 9 | Does it describe how each treatment works? | 1-5 |
|  | 10 | Does it describe the benefits of each treatment? | 1-5 |
|  | 11 | Does it describe the risks of each treatment? | 1-5 |
|  | 12 | Does it describe what would happen if no treatment is used? | 1-5 |
|  | 13 | Does it describe how the treatment choices affect overall quality of life? | 1-5 |
|  | 14 | Is it clear that there may be more than 1 possible treatment choice? | 1-5 |
|  | 15 | Does it provide support for shared decision making? | 1-5 |
| Section 3 | 16 | Based on the answers to all of these questions, rate the overall quality of the publication as a source of information about treatment choices. | 1-5 |

**Supplementary Table 1**. DISCERN scoring system.
