# Supplementary material for: Quality of Internet Videos Related to Pediatric Urology in Mainland China: A Cross-Sectional Study
Source: Front Public Health. 2022 Jun 15;10:924748. doi: 10.3389/fpubh.2022.924748 (PMC9240759; doi:10.3389/fpubh.2022.924748)
Supplement: Supplementary Table 2 — JAMA benchmark criteria. [file Table_2.DOCX]

**Supplementary Table 2**. JAMA Benchmark Criteria.

| JAMA Benchmarks | Explanation | Score |
| --- | --- | --- |
| Authorship | Authors and contributors, their affiliations, and relevant credentials should be provided | 0 or 1 |
| Attribution | References and sources for all content should be listed clearly, and all relevant copyright information should be noted | 0 or 1 |
| Disclosure | Website “ownership” should be prominently and fully disclosed, as should any sponsorship, advertising, underwriting, commercial funding arrangements or support, or potential conflicts of interest | 0 or 1 |
| Currency | Dates when content was posted and updated should be indicated | 0 or 1 |
