# Supplementary material for: Quality of Internet Videos Related to Pediatric Urology in Mainland China: A Cross-Sectional Study
Source: Front Public Health. 2022 Jun 15;10:924748. doi: 10.3389/fpubh.2022.924748 (PMC9240759; doi:10.3389/fpubh.2022.924748)
Supplement: Supplementary Table 3 — Hexagonal charting system. [file Table_3.DOCX]

**Supplementary Table 3**. Hexagonal Charting System.

| Category | Criteria | Score |
| --- | --- | --- |
| Definition | Defining a disease or a particular stage, type, category of the disease | Not addressed at all = 0  Slightly addressed = 0.5  Partially addressed = 1  Quite well addressed = 1.5  Fully addressed = 2 |
| Signs | Typical signs caused by the disease |  |
| Risk factors | Factors that might cause the incidence of the disease or accelerate its progress |  |
| Examination | Means used for diagnosing and evaluating the disease |  |
| Management | Treatment |  |
| Outcomes | Prognosis of the disease, complications, survival or death |  |
